# Implementation of a Social Media Strategy for Public Health Promotion in Black, American Indian or Alaska Native, and Hispanic or Latino Communities During the COVID-19 Pandemic: Cross-Sectional Study

**DOI:** 10.2196/58581

**Published:** 2024-12-10

**Authors:** Maria Mora Pinzon, Ornella Hills, George Levy, Taryn T James, Ashley Benitez, Sacheen Lawrence, Tiffany Ellis, Venus Washington, Lizbeth Solorzano, Patricia Tellez-Giron, Fernando Cano Ospina, Melissa F Metoxen, Carey E Gleason

**Affiliations:** 1 Division of Geriatrics and Gerontology, Department of Medicine School of Medicine and Public Health University of Wisconsin - Madison Madison, WI United States; 2 Department of Ethnic Studies College of Arts and Sciences Lawrence University Appleton, WI United States; 3 Wisconsin Alzheimer’s Disease Research Center, Department of Medicine, School of Medicine and Public Health University of Wisconsin – Madison Madison, WI United States; 4 School of Medicine and Public Health, University of Wisconsin – Madison Madison, WI United States; 5 Venus Inspires Madison, WI United States; 6 UnityPoint Health - Meriter Madison, WI United States; 7 Latino Health Council of Dane County Madison, WI United States; 8 Native American Center for Health Professions, School of Medicine and Public Health University of Wisconsin - Madison Madison, WI United States; 9 Geriatric Research, Education and Clinical Center, William S. Middleton Memorial Veterans Hospital, Department of Veterans Affairs Madison, WI United States

**Keywords:** health communications, social media, Hispanic, Latino, Black, American Indian, Alaska Native, minority health, health disparities, COVID-19

## Abstract

**Background:**

Individuals identifying as Black, American Indian or Alaska Native, or Hispanic or Latino lack access to culturally appropriate accurate information and are the target of disinformation campaigns, which create doubt in science and health care providers and might play a role in sustaining health disparities related to the COVID-19 pandemic.

**Objective:**

This study aims to create and disseminate culturally and medically appropriate social media messages for Black, Latino, and American Indian or Alaska Native communities in Wisconsin and evaluate their reach and effectiveness in addressing the information needs of these communities.

**Methods:**

Our team identified relevant COVID-19 topics based on feedback from their respective community, developed lay format materials, and translated materials into culturally appropriate social media messages that community advocates delivered across their respective communities. Social media metrics (reach, engagement, and impressions) were collected using Sprout Social and Facebook Analytics. We hosted 9 focus groups with community members to learn about their social media use. These data were analyzed using an inductive approach, using NVivo software (release 1.7) to code content.

**Results:**

Between August 2021 and January 2023, we created 980 unique social media posts that reached 88,790 individuals and gathered >6700 engagements. Average reach per post was similar across the 3 communities, despite differences in the number of posts and followers on each page: 119.46 (Latino individuals), 111.74 (Black individuals), and 113.11 (Oneida Nation members). The type of posts that had higher engagement rate per reached person (ERR) varied across communities and platforms, with the highest being live videos for the Latino community on Facebook (ERR 9.4%), videos for the Black community on Facebook (ERR 19.53%), and social media messages for the Oneida Nation community (ERR 59.01%).

**Conclusions:**

Our project presents a unique and effective model for health messages and highlights the need for tailoring social media messages and approaches for minoritized audiences (eg, age, gender, race, and ethnicity). Further research studies are needed to explore how specific types of information affect the dissemination of information and the implications for health communications.

## Introduction

### Background

Individuals from Black, American Indian or Alaska Native, and Hispanic or Latino communities were disproportionately impacted by the SARS-CoV-2 virus that caused the COVID-19 pandemic. Multiple studies reported that Black, Hispanic, and American Indian or Alaska Native communities had higher hospitalization rates and death rates across the country [1,2]. In Kaiser Family data samples from >40 states, it was found that the rate of infection for Hispanic patients was >3 times the rate for White patients, and from March to July 18, 2020, hospitalization rates due to the COVID-19 pandemic for Black, American Indian or Alaska Native, and Hispanic patients were approximately 5 times higher than those for White patients [3,4]. In Wisconsin, compared with non-Hispanic White Wisconsin residents, Hispanic or Latino residents had 1.7 times higher case rates, Black residents had 2.1 times higher hospitalization rates, and American Indian residents had 1.4 times greater death rates [5].

Greater prevalence of comorbid conditions, such as diabetes, obesity, and cardiovascular disease, among Black, American Indian or Alaska Native, and Hispanic or Latino patients elevated their risk of hospitalization once infected [1]. Poor access to health care, low physician trust delaying help seeking and systemic racism and mistreatment in medical care likely worsened these hospitalization rates and death rates [1]. Furthermore, individuals who identify as Black, American Indian or Alaska Native, or Hispanic or Latino are more likely to live in socioeconomically disadvantaged neighborhoods [6,7], tend to rely more on public transportation, are more likely to live in multigenerational households, and are more likely to hold jobs that cannot be performed remotely [1,6], leaving them at a higher risk of exposure to SARS-CoV-2 virus. Notably, McCormack et al [8], in their study of >3 million persons, found that Black individuals were overrepresented in multiple industries with essential workers, including transportation, public administration, and health care. In many cities, these neighborhoods with communities of color have been segregated, which increased the concentration and spread of the virus in these neighborhoods and likely isolated its impact to these racially segregated communities [6]. The disparities in vaccination rates only exacerbated the issue. By May 25, 2021, a total of 44% of White residents of Wisconsin had received at least 1 dose of the COVID-19 vaccine. However, only 23% of Black residents, 29% of American Indian residents, and 31% of Hispanic or Latino residents had received a dose [9]. The reasons for lower vaccination rates were varied, including systemic racism [10], disparities in accessing care, lack of information about vaccination centers, misinformation and disinformation campaigns [11], and confusion about benefits and eligibility criteria [12].

During the COVID-19 pandemic, the lack of in-person interactions inhibited the use of traditional outreach approaches to address the misinformation, resulting in an increased use of social media [13]. As more individuals spend more time on online social networks, information and misinformation spread farther and faster than ever before, requiring the development of new approaches for using social media for public health promotion [14,15]. These approaches also needed to account for the mistrust in governmental and research organizations that communities of color might experience due to the structural racism, historical inequities, language of preference, and health literacy of the communities.

In light of the misinformation campaigns that were targeting communities of color [16], the community advisory board of the Wisconsin Alzheimer’s Disease Research Center made the recommendation that the team pivot and focus on providing accurate information to communities of color on the COVID-19 pandemic. After exploration with additional community partners, there was a high level of interest in a social media solution that sought to address the needs of Black, Latino, and American Indian or Alaska Native communities in the state of Wisconsin by creating culturally appropriate content that promotes medically accurate information about the COVID-19 pandemic.

Multiple psychological theories, such as cultivation theory [17] and attribution theory, can be used to explore how media messages, including social media messages, affect individuals’ perceptions and behaviors. Cultivation theory suggests that long-term exposure to media content shapes an individual’s beliefs, attitudes, and perceptions about the world. It argues that heavy viewers of television, for example, are more likely to adopt the values and perspectives presented in media programming, leading to a *cultivation* of their worldview [17]. In the context of social media, for example, if social media messages were to consistently portray smoking as a common and acceptable behavior, individuals who are heavy users of social media may develop a skewed perception of smoking norms, potentially impacting their attitudes and behavior regarding smoking cessation or prevention [18]. Therefore, by strategically incorporating positive health messages in social media content, public health campaigns can leverage the cultivation theory to influence people’s beliefs and behaviors in favor of healthier choices.

Increasingly, more Americans use social media platforms, according to a Pew Research Center Study in 2021, with vast demographic differences per platform [19]. A total of 81% of all respondents used YouTube (Google LLC) while 95% of adults aged 18 to 29 years used the platform. The study also reported racial and ethnic differences. Higher rates of WhatsApp (Meta Platforms, Inc) use were reported among Hispanic respondents, with 49% of them using WhatsApp compared with only 16% of White respondents. A total of 49% of Black respondents used Instagram (Meta Platforms, Inc) and 74% used Facebook compared with only 35% of White residents using Instagram and 67% of them using Facebook. Given that misinformation is reported to be more easily spread via social media [20] and Black, American Indian or Alaska Native, and Hispanic or Latino communities report heavier use of social media, public health professionals could harness cultivation theory effects to increase positive social media messaging that combat misinformation in social networks frequented by Black, American Indian or Alaska Native, and Hispanic or Latino individuals.

Attribution theory [21] focuses on how individuals attribute causes to the behavior of themselves and others. It suggests that people tend to make inferences about the underlying causes of behavior based on 3 main dimensions: internal factors (eg, personal characteristics), external factors (eg, situational factors), and stability (eg, whether the behavior is consistent or temporary). In public health messaging, attribution theory can play a role in understanding how people interpret health-related behaviors and social media messages shared on social media. Individuals may attribute the behavior of others, such as engaging in health activities or practicing preventive measures, to internal factors (eg, personal motivation or responsibility) or external factors (eg, societal norms of situational influences) [22]. Public health campaigns can leverage the attribution theory by highlighting positive health behaviors and framing them in a way that encourages attributions to internal factors (eg, personal agency) to motivate individuals to adopt healthier behaviors themselves.

Together, cultivation and attribution theories reflect how health professionals can develop more effective social media strategies to engage and influence their audience’s health behaviors and shape their perceptions related to health issues.

### Objectives

This implementation report aimed to describe the process used to develop social media content and deploy multimedia strategies to communicate with 3 different populations: Black, American Indian or Alaska Native, and Hispanic or Latino communities on digital platforms, as guided by these psychological frameworks. Furthermore, we present data from a comprehensive evaluation of our processes’ effectiveness. Specifically, we provide quantitative data obtained from social media metrics and qualitative data obtained from focus groups with community members from the 3 communities. This information provides evidence that addresses some of the gaps in knowledge about using social media in health promotion [13] and provides a blueprint for other organizations seeking to partner with communities of color to address the community’s informational needs.

## Methods

### The Communications Campaign

#### Team Formation

The core team of this project consisted of a media specialist with experience in digital campaigns, an expert on mass communications for communities of color, and community members to serve the role of community influencers (later self-renamed as community advocates). The community advocates did not need to have experience in using social media, and the main requirement was to have connections within their self-identified community and a desire to share information with their community. For the Latino community, we also partnered with members of the Latino Health Council of Dane County (a nonprofit organization that convenes organizations providing services to Latino communities), who created and disseminated additional content. As the project evolved, team members were added to the core team to provide expertise and assistance in translating scientific findings into layperson language, including a team scientist who belongs to one of the communities of interest with experience in presenting scientific information to lay audiences and health care professionals (eg, emergency medicine physicians, medical students, and other health professionals).

#### Platforms and Pages

To reach the Black community in Wisconsin, in August 2020, we created a Facebook (Meta Platforms, Inc) page titled “COVID-19 Information for the Black Community” [23], and in January 2022, we created an Instagram (Meta Platforms, Inc) professional account titled @blackwellness. For reaching members of the Oneida Nation, a federally recognized American Indian tribe in Wisconsin, we created a Facebook page titled “COVID-19 Information for the Oneida Nation” [24], which was usually shared or reshared by the Oneida Nation’s official Facebook page (that has >14,000 followers). For the Latino community, all the posts and content were disseminated through the social media channels of the Latino Health Council of Dane County (Twitter [Twitter, Inc; subsequently rebranded as X, X Corp], Instagram, Facebook [25], and YouTube [Google LLC]); this organization was selected based on long-standing partnerships and the intention to synergize with existing education initiatives around the COVID-19 pandemic that they were implementing. The selection of the platforms was informed by the community advocates and based on the use of the platform by each community in the United States from 2020 to 2021, which, according to Pew Research Center, were Facebook and YouTube, with an increased use of Instagram by those aged <30 years [19].

#### Selection of Topics

Monthly meetings were scheduled with the principal investigators (MM, MMP, and CEG) and the team to define the topics, which were based on upcoming national and cultural holidays (eg, Indigenous Peoples’ Day and Dia de los Muertos), relevant events (eg, the start of the school year and vacations), and new developments related to the COVID-19 pandemic (eg, new recommendations from the Centers for Disease Control and Prevention). The advocates’ assessment of their communities’ most pressing concerns was the primary driver for the topics selected.

#### Development and Dissemination of Content

The core team met weekly to prepare posts, evaluate performance, and develop new communication strategies. Advocates were asked to post content weekly on their platform of choice. Advocates were never required to post on topics to propagandize or promote a specific agenda. Developing content was iterative but started with creating a scientifically informed response to the topic. To support their posts, the group, including physicians and other professionals, met weekly to clarify information and ensure that the content was medically accurate, culturally relevant, and evidence based. This often included a weekly presentation by our team scientist (TTJ) on the week’s topics to address gaps in knowledge of the scientific materials prepared in layperson format. The team scientist stayed abreast of local and national news on the COVID-19 pandemic and pertinent health topics and searched social media spaces targeted to communities of color to understand myths and disinformation surrounding the COVID-19 pandemic to better serve the communities. All team members had to feel comfortable with the content being disseminated as it was to ensure authenticity.

**Figure 1:**
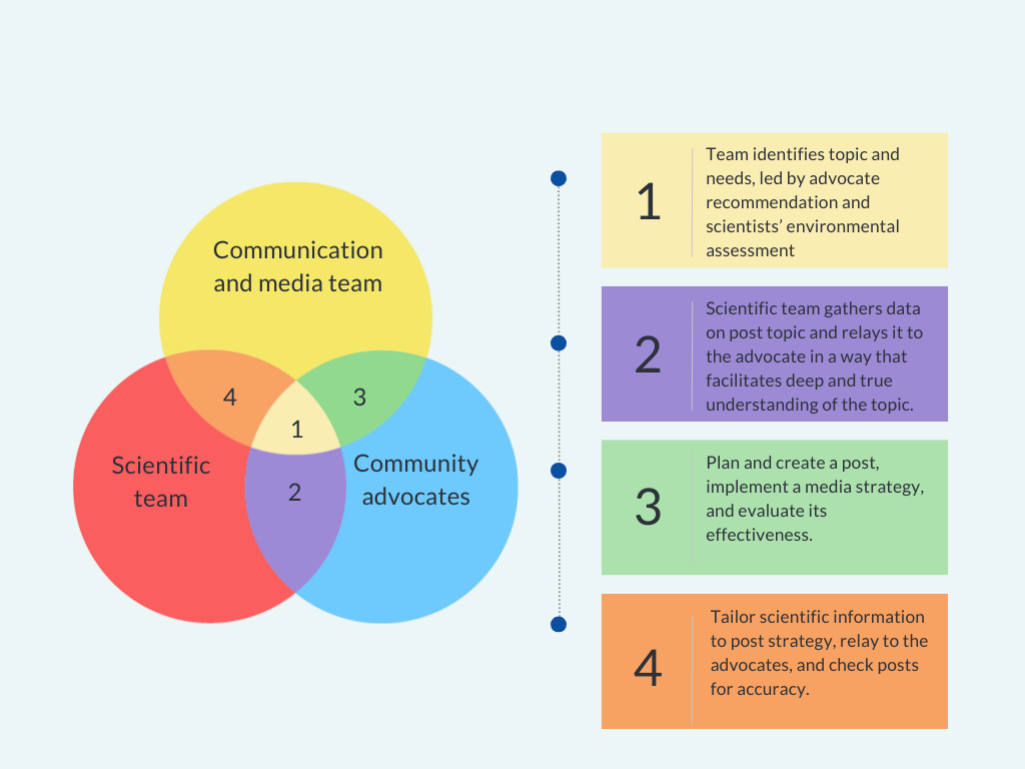
Graphical representation of the content creation process, including team roles and responsibilities.

Finally, the community advocates (TE, AB, LS, VW, and SL) drafted their posts using this rapport. However, they were directly supported by the communications team to create graphics and tailor the social media message to align with the individual community. Community advocates also received training on communication tools and resources to improve their content (eg, video editing and creating infographics). Figure 1 shows a graphic representation of the process used to develop content and the people involved in each step of the process.

Some of the social media messages posted included how vaccines work, updates on approvals of vaccines, correct use of masks, mental health checkups, and interviews with experts (Multimedia Appendix 1). Advocates posted content on the platforms directly or through a social management platform. The hashtags used and the day and time of the posting were agreed upon with the communications team based on the performance of previous posts. We did not use paid advertising or boosts to disseminate the social media messages.

### Data Collection and Analysis

#### Quantitative Data

We evaluated the success of our digital media efforts by monitoring reach and engagements. We used the analytic tools provided by the following platforms: Facebook and Sprout Social (Sprout Social, Inc). In March 2023, once the study was completed, we downloaded analytic reports for each community page’s study period. Because the Latino pages included content created by additional volunteers and other sources, we selected a random sample of posts (1 per week during the study period) to collect the analytics of interest in the study.

The metrics of interest in this study are defined as follows:

1. *Reach*: the total number of unique individuals who see the content from the account
2. *Engagements*: unique individuals engaged with the content, either by liking the post, following the page or account, sharing the content, or clicking one of the links in the post.

Because metrics related to reach and engagement are dependent on the number of followers per page, we analyzed 2 rates to be able to compare the posts’ performance across different communities and platforms: engagement rate per reached person (ERR, number of engagements divided by the number of persons reached) and average reach per post (ARPP, number of persons reached divided by the number of posts). We obtained the 95% CI for these rates using the MedCalc (MedCalc Software Ltd) software [26]. We conducted comparative analysis of the ERR according to the post type (link, live, photo or image, text, and video); and language (bilingual, English, and Spanish). Statistical significance was assessed using *P* values, which were calculated using chi-square tests or Fisher exact tests, where values <.05 were considered statistically relevant. Our results are presented separately for each community and platform, and no direct comparisons were made across communities.

#### Qualitative Data

Data were collected through 9 focus groups (3 per community). The interview guide was developed by the community advocates, communication specialist, and author MMP (coprincipal investigator) to ensure that the questions addressed the main concerns of the team. The interview guide was revised by CG and MM (coprincipal investigators) and further edited by experts in qualitative approach who facilitated the focus groups.

Participants in the focus groups were recruited through flyers and short videos the advocates posted on our social media platforms. In addition, we asked partner community organizations to share our posts to increase the reach and share via other platforms used by the community (eg, group chats).

Interested individuals were provided with a link, which directed them to a REDCap (Research Electronic Data Capture; Vanderbilt University) electronic data form hosted at the University of Wisconsin–Madison Department of Medicine [27]. The form collected information to assess eligibility for participation (eg, age, race or ethnicity, and use of social media platforms) and contact information.

Eligibility criteria included self-identifying as a Black or African American, American Indian or Alaska Native, or Hispanic or Latino individual; being aged >18 years; and having self-reported awareness of the community pages created for this study. For one of the focus groups of each community, we only included participants who reported following the social media page of interest.

The focus groups were audio recorded, and verbatim transcripts were made from the audio recordings, ensuring that these were deidentified for analysis. Data were analyzed using an inductive approach to code the transcripts, and the themes in this report were generated from participants’ answers rather than defined in advance. Coding was done with NVivo (release 1.7; Lumivero).

### Ethical Considerations

This study was not considered human subject research by the University of Wisconsin–Madison Institutional Review Board and was classified as quality improvement or program evaluation not requiring an institutional review board approval in accordance with federal regulations, as defined under 45 Code of Federal Regulations Part 46.102(d). This report follows the recommendations of the iCHECK-DH (Guidelines and Checklist for the Reporting on Digital Health Implementations) [28] guidelines, and the checklist is available in Multimedia Appendix 2.

The research team contacted individuals to obtain informed consent and provide focus group details. During the introduction of the section, participants were reminded about the volunteer nature of participation and provided additional opportunities to opt out. Participants received US $60 to US $75 as a token of appreciation for participating in the 90-minute focus group; the specific amount changed across the communities based on the timing of the recruitment.

## Results

### Use of Social Media

#### Overview of Focus Groups

A total of 9 focus groups were hosted across the 3 communities; 7 (78%) were via videoconferencing (Zoom; Zoom Video Communications), and 2 (22%) were conducted in person. Among a total of 59 individuals who participated, 45 (76%) were women and 19 (32%) self-identified as members of the Oneida Nation, 18 (30%) as Black or African American individuals, and 21 (36%) as Hispanic or Latino individuals. Classification according to the age group of participants is as follows: 17% (10/59) were aged 18 to 24 years, 20% (12/59) were aged 25 to 34 years, 31% (18/59) were aged 35 to 44 years, 9% (5/59) were aged 45 to 54 years, and 21% (12/59) were aged >55 years.

The following paragraphs reflect participants’ responses to different topics. Where dialogue is needed to convey context or meaning, focus group participant and moderator responses are indicated by “P” and “M,” respectively. For quotations that stand alone (ie, without dialogue or a speaker tag such as “P”), it can be assumed that the speaker is a focus group participant. The racial or ethnic identity of the focus group participant is indicated at the end of the quotation with a parenthetical code that includes the participant’s race or ethnicity and focus group number (Black or African American, Oneida Nation, or Hispanic or Latino individual).

#### Reasons to Use of Social Media

Participants liked using social media to keep up with people they knew, as stated as follows:

I like seeing other people’s pictures, and what they’re up to, how they’ve been.

Oneida Nation individual, 1

I keep in touch, I have different kinds of groups I have. I have my high school folks that I keep in touch with. I have my folks that I went to [college name] with. I have my church family. I have friends that I went to school with.

Black or African American individual, 1

I use Facebook, but more for its Marketplace or groups that I follow. It’s also the only way I can see a certain part of my family.

Hispanic or Latino individual, 1

Participants liked and shared various posts, including memes, posts that make them laugh, posts about sports, and community events. Some participants talked about using social media to obtain information about the COVID-19 pandemic:

I don’t really watch the news, so I use it a lot for keeping up with information about COVID, different health things with COVID, keeping up with the numbers of COVID, the guidelines, the things that I should and shouldn’t be doing as far as COVID.

Black or African American individual, 1

I kind of just look at the page for myself to keep myself up to date.

Black or African American individual, 3

I usually share a lot of information about benefit to the community. So, I serve as a source of publicity, so to speak, or have served my family.

Hispanic or Latino individual, 1

#### Kinds of Posts That Participants Disliked

One thing many participants did not like about Facebook in particular was the number of unsolicited advertisements on their wall; they usually ended up blocking them even when they may be of interest. They felt that these advertisements were intrusive:

What I don’t like is that messages appear that no one is asking for... I really have to be blocking many messages that are not what I have looked for.

Hispanic or Latino individual, 1

Similarly, individuals were asked what type of information or strategy would make posted content more persuasive, and they expressed that a lack of authenticity from the author would decrease the likelihood of changing their existing beliefs.

...that post actually sounds like that lady was paid to post that. So that wouldn’t change my mind to... I mean, it just sounds too— I don’t know, it just sounds too payish, like she got paid to write it, like it was supposed to sound perfect.

Black or African American individual, 2

### Overall Use and Engagement Metrics

**Figure 2:**
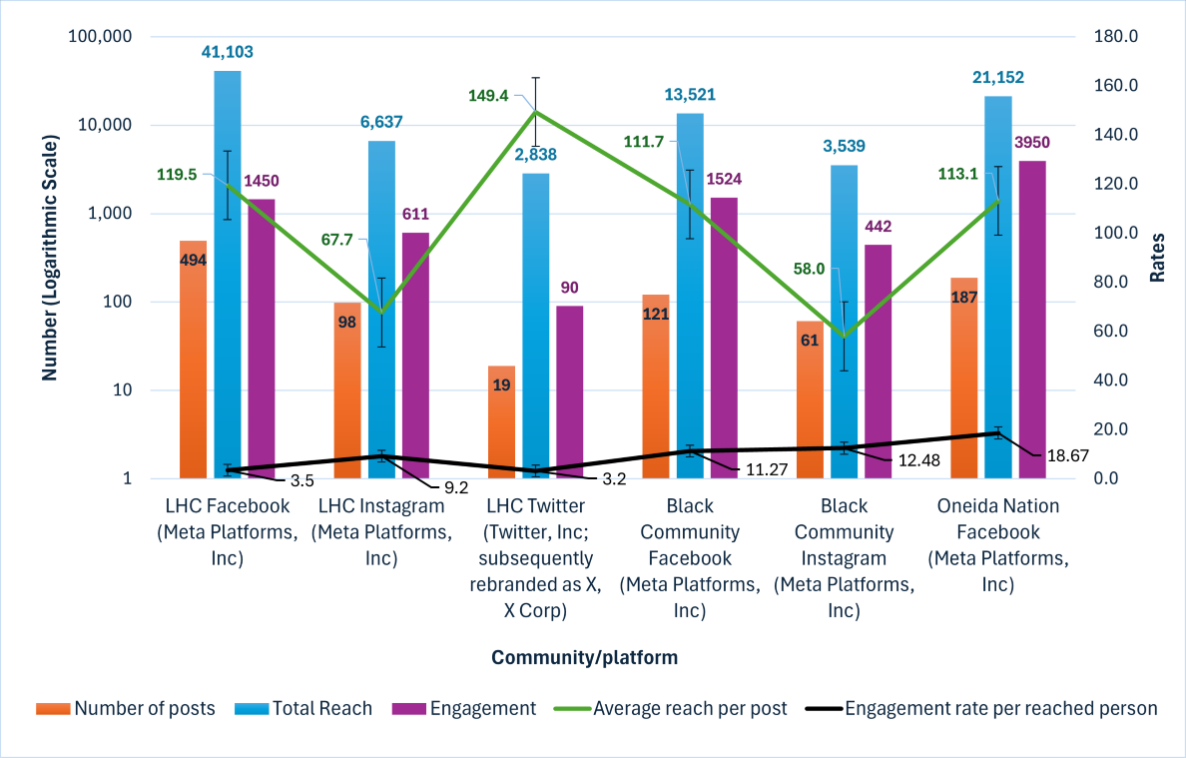
Overall performance of posts according to community (July 2021 to January 2023).

Between August 2021 and January 2023, we created 980 unique social media posts that reached 88,790 individuals and gathered >6700 engagements. Figure 2 describes the overall number of posts per community and platform and their associated social media metrics. Posts directed to members of the Black community showed an ARPP comparable with the other Facebook communities in this project (111.74 for Black or African American vs 119.46 for Hispanic or Latino vs 113.11 Oneida Nation) despite the lowest overall post count. Within the Latino community, Instagram posts had a higher ERR (9.2% vs 3.5% vs 3.2%; *P*<.001) despite the lower number of posts and reach. Multimedia Appendix 3 shows the demographics of the followers of each of the community platforms.

Some participants reported that they might not engage with a post because they want to avoid notifications or well-intended inquiries from others who can see their interaction.

I think, for me, if I were feeling anxiety or depressed or something, I probably would not. But if I was in a good mood, I’d probably like it or send a heart. Because... if you feel something that’s down, you’re going to get people saying, are you okay, or calling you or texting you, and I don’t know that I’m always ready for that. People read into your reaction sometimes. I think about what others think. I know that. So sometimes I may not react at all because I don’t want feedback.

Black or African American individual, 1

### Reach and Engagement of Posts for Latino Communities

#### Overview

Table 1 presents the social media metrics according to the post type per platform. On Facebook, live videos had the highest ARPP and ERR (*P*<.001), while posts that comprised only social media messages had the lowest engagement. Meanwhile, on Instagram, videos had the highest ARPP (94.07) and photos or images had the highest ERR (10.17%; *P*=.002).

Focus group participants mentioned that combining graphics or images with text is more effective at catching the attention of Hispanic or Latino individuals rather than just catchy phrases with large fonts:

I am quite a very visual person. If I see this photo, it catches my attention and I say, if they went [to get vaccinated], why don’t I go? So, our Latino community is quite visual, if you put a photo, it will reach you much more than put some big letters... That is to say, everything that we put in pure letters does not reach as much as a photo.

Hispanic or Latino individual, 1

**Table 1:** Metrics for Latino community platforms according to the type of posts (August 2021 to January 2023).

Community			Posts, n (%)		Total reach, n		Total engagement, n		ARPP^a^ (95% CI)		ERR^b^ (%; 95% CI)		*P* value
**Facebook (n=135)**													
	Link	29 (21.5)		2306		193		79.52 (76.31-82.83)		8.37 (7.23-9.64)		<.001	
	Live	13 (9.6)		3871		364		*297.77 (288.46-307.3)* ^c^		*9.40 (8.46-10.42)*		—^d^	
	Photo or image	76 (56.3)		11,734		454		154.39 (151.61-157.21)		3.86 (3.52-4.24)		—	
	Text only	2 (1.5)		300		9		150 (133.5-167.97)		3.00 (1.37-5.69)		—	
	Video	15 (11.1)		1471		53		98.07 (93.12-103.21)		3.60 (2.69-4.71)		—	
**Instagram (n=98)**													
	Photo or image	70 (71.4)		4003		407		57.18 (55.43-58.99)		*10.17 (9.2-11.2)*		.002	
	Video	28 (28.6)		2634		204		*94.07 (90.51-97.73)*		7.75 (6.72-8.88)		—	

^a^ARPP: average reach per post.

^b^ERR: engagement rate per reached person.

^c^Italicized values indicate the highest category in the group.

^d^Not applicable.

#### Language

Table 2 shows that on Facebook, posts that were only in English had the largest ARPP, while those in Spanish had the highest ERR (6.99%; *P*<.001). On Instagram, there was no statistical difference in ARPP and ERR according to language (*P*=.10).

In contrast, focus group participants found that posts in both languages were more impactful because they were more accessible to a larger audience. Some participants felt that it is a misconception that to be a Latino, you have to speak Spanish; many individuals in the younger generations of Latinos have lost fluency of the Spanish Language and are uncomfortable with being excluded because of it. In contrast, many felt it harder to translate English-only posts to friends and family, as information is lost in translation. Many people found sharing and discussing content with family more appealing when there was a picture and content in English and Spanish:

Personally, I like it [bilingual posts] because I find it accessible. That is because perhaps there are terms that I do not handle in one language, and in another [language], that same term has more context. And that makes me able to use both languages to better understand the content.

Hispanic or Latino individual, 1

**Table 2:** Metrics for Latino community platforms according to the language of posts (August 2021 to January 2023).

Platform			Posts, n (%)		Total reach, n		Total engagement, n		ARPP^a^ (95% CI)		ERR^b^ (%; 95% CI)		*P* value
**Facebook (n=135)**													
	Bilingual	6 (4.6)		519		31		86.5 (79.22-94.27)		5.97 (4.06-8.48)		<.001	
	English	28 (21.4)		5029		40		*179.61 (174.68-184.64)* ^*c*^		0.79 (0.56-1.08)		—^d^	
	Spanish	97 (74)		11,818		827		121.84 (119.65-124.05)		*6.99 (6.53-7.49)*		—	
**Instagram (n=98)**													
	Bilingual	31 (31.6)		2544		249		82.07 (78.91-85.32)		9.79 (8.61-11.08)		.10	
	English	26 (26.5)		2203		177		*84.7 (81.23-88.35)*		8.03 (6.89-9.31)		—	
	Spanish	41 (41.8)		1890		185		46.09 (44.04-48.22)		*9.79 (8.43-11.31)*		—	

^a^ARPP: average reach per post.

^b^ERR: engagement rate per reached person.

^c^Italicized values indicate the highest rate in the category.

^d^Not applicable.

### Reach and Engagement of Posts for Black Communities

Table 3 presents the social media metrics according to the type of posts of each platform for the Black community. On Facebook, posts with photos had the largest ARPP (132.85), while posts containing only text had the lowest; videos had the largest ERR (19.53%; *P*<.001). For Instagram, videos had the largest ARPP, and carousel posts had the largest ERR (*P*<.001).

**Table 3:** Metrics for platforms for the Black community according to the type of post (August 2021 to January 2023).

Type of post			Posts, n (%)		Total reach, n		Total engagement, n		ARPP^a^ (95% CI)		ERR^b^ (%; 95% CI)		*P* value
**Facebook (n=121)**													
	Link	19 (15.7)		657		83		34.58 (31.98-37.32)		12.63 (10.06-15.66)		<.001	
	Photo or image	74 (61.2)		9831		851		*132.85 (130.24-135.5)* ^*c*^		8.65 (8.08-9.25)		—^d^	
	Text only	2 (1.7)		23		2		11.5 (7.29-17.26)		8.69 (1.05-31.4)		—	
	Video	26 (21.5)		3010		588		115.77 (111.67-119.98)		*19.53 (17.99-21.18)*		—	
**Instagram (n=61)**													
	Carousel	11 (18)		426		85		38.73 (35.14-42.59)		*19.95 (15.94-24.67)*		<.001	
	Photo or image	22 (36.1)		919		118		41.77 (39.12-44.56)		12.84 (10.63-15.38)		—	
	Video	28 (45.9)		2194		239		*78.36 (75.11-81.71)*		10.89 (9.56-12.37)		—	

^a^ARPP: average reach per post.

^b^ERR: engagement rate per reached person.

^c^Italicized values indicate the highest rate in the category.

^d^Not applicable.

All participants reacted favorably to the example video played [29]. Participants cited the following reasons why this video caught their attention:

- It uses a popular song:

It’s a Beyonce song. And then I’m going to automatically read the information, and then I’m going to sing the song while I’m at it.

Black or African American individual, 1

- It was funny:

I got a good laugh out of it

Black or African American individual, 1

- It feels comfortable and culturally familiar:

It’s easily hearable because like as for me and my house, like it feels familiar. It feels comfortable.

Black or African American individual, 1

I like to see things that appeal to me and my culture.

Black or African American individual, 1

### Reach and Engagement of Posts for the Oneida Nation Page

Table 4 shows the social media metrics for the Facebook page “COVID-19 Information for Oneida Nation Community” according to the post type. Video posts had an ARPP that was statistically significantly larger than other posts (179.27; *P*<.001), and the largest ERR was seen on posts that had only text.

All Oneida Nation members who participated in the focus groups were positive about posts that used images or videos. Several participants said they preferred videos that were ≤2 minutes in length, with 1 participant checking the video length first, skipping portions of long videos, and watching just the ending:

P7: ...like tops that I want to watch a video for is maybe like 1.5 or 2 minutes if it’s something good....

P8: I would say 2 minutes....

M: Okay. Why 2 minutes?

P8: It’s short and to the point.

P6: I’d say like a minute or 2 minutes, yeah, like everybody else was saying. Just get to the point, you know. Nobody really wants to sit there and have to scroll through or just watch it, you know. Really, if you really could just have it short, get to the point, it will be more interesting. More people will probably watch it....

P5: I’d say 1-2 minutes as well.

Oneida Nation individual, 1

Participants stated that it is especially important that a video be short if it is a topic that people are not following or not already interested in:

If it’s something like, you know, you actually like, I mean, I guess you’re going to watch it no matter how long it’s going to be. But if it’s something that you really don’t like, or you’re not really interested in, I guess you’re not going to have no—you know, you’re not going to waste time and watch it.

Oneida Nation individual, 1

**Table 4:** Metrics for the Facebook (Meta Platforms, Inc) page for the Oneida Nation according to the type of post (August 2021 to January 2023).

Post characteristic	Posts (n=187), n (%)	Total reach, n	Total engagement, n	ARPP^a^ (95% CI)	ERR^b^ (%; 95% CI)	*P* value
Link	21 (11.2)	64	846	40.3 (37.6-43.09)	7.57 (5.82-9.66)	<.001
Photo or image	102 (54.5)	1509	9398	92.13 (90.28-94.02)	16.06 (15.3-16.89)	—^d^
Text only	10 (5.3)	724	1227	122.7 (115.93-129.76)	*59.01 (54.79-63.46)* ^*c*^	—
Video	54 (28.9)	1653	9681	*179.27 (175.72-182.89)*	17.07 (16.26-17.92)	—

^a^ARPP: average reach per post.

^b^ERR: engagement rate per reached person.

^c^Italicized values indicate the highest rate in the category.

^d^Not applicable.

### Perception of the Community Individuals to Our Social Media Campaigns

Everyone familiar with the COVID-19 Info for Madison’s Black Community Facebook page praised it for having reliable and relatable information:

It’s informational and it’s easy to read and to understand what’s there, which is part of what I think a lot of the issue is, is people don’t necessarily like reading. And so the graphics and the wording is very clear, and anybody can understand it, no matter what their educational background is.

Black or African American individual, 1

Similarly, there was uniform favorability toward posts that offered resources and ideas to promote health. Participants cited the benefits to themselves, their families, and their communities:

I think they’re helpful resources. They’re just there, you know. You can take a look, take a glance. If you do need that type of information, then it’s there for you to get it open to the public, you know, good information.

Oneida Nation individual, 1

It’s kind of like you hear on the news, like when they have recalls for food and stuff, it’s like very informational things that I would like to know. So, I mean, everybody kind of benefits from posts like that.

Oneida Nation individual, 1

## Discussion

### Principal Findings

Our study shows that social media communications for health promotion were favorably perceived by individuals from Black, American Indian or Alaska Native, and Hispanic and Latino communities when produced by reliable sources with whom they are already connected in real life. Similarly, there is evidence that social network sites are becoming essential to how people experience news, moving from a traditional news cycle that journalists primarily dominate to more complex information cycles that incorporate ordinary people within the process [30].

In our study results, individuals reflected that social media platforms are used for recreational purposes and staying up to date with family and friends. Prior research indicates that individuals from marginalized communities also use social media to find health information [31,32]. Given that racial and ethnic minority groups use these media platforms more than White individuals [13], our study developed processes to meet these populations on digital platforms in a way that promoted trusted and accurate dissemination of public health information. This model is distinct from prior studies using social media influencers as the community influencers were not merely tools for disseminating public health social media messaging created by officials and scientists [33-35].

As described in the *Methods* section, our community influencers determined what content was relevant to their communities and were empowered through skill building to be codrivers at every stage of the layperson translation process, from strategy to content creation to dissemination and analysis. This approach is similar to previous descriptions of tailored influencer marketing approaches that harness the social influencers’ experiences and competencies [36]. An additional key distinguishing feature of our model is detailed knowledge sharing between the scientists and the community influencers, which led to the self-renaming of the influencers to advocates as they began to take ownership and gain a proper understanding of the scientific content being shared on their pages. According to the attribution theory, these interactions within our team affected how the information was presented and ensured that the most relevant information for the audience was presented first, which might, in turn, influence the audience’s perception of the stability and controllability of health information, leading to a higher likelihood of message acceptance and behavior change.

Our results also highlight that there is not 1 unique strategy that works across various Black, American Indian, Alaska Native, and Hispanic or Latino communities. Health promotion social media messages must be tailored to the audience of interest and the platform used. Facebook audiences were mostly middle-aged individuals, while Instagram had more young adults and teenagers, which is consistent with the existing literature [37]. The Instagram account also reached a higher percentage of men than Facebook and even reached other communities of color in low- and middle-income countries (Colombia, India, Mexico, Nigeria, and Venezuela).

To increase the dissemination of social media messages, these must be easy to understand and attention grabbing to generate interest in audiences while being authentic without creating the sensation of spreading propaganda. The most successful strategies we identified to accomplish these goals were live streaming and videos. Videos had the advantage of overcoming literacy limitations, and when they displayed people, these created a sense of connection and authenticity with the audience, which partially addressed one of the limitations of social media: the lack of reliable sources [13]. These results are similar to those obtained by other studies that reflect that the content needs to be attractive and easy to understand to be effective [38].

The focus group data indicated that people use social media to keep in touch with people they already know or are connected to, which suggests that for future endeavors, it is paramount to identify an advocate who is highly connected to their community, as the audiences would be more likely to pay attention and trust the information presented.

Unsurprisingly, given the historical abuse endured by marginalized communities, trust and authenticity emerged as critical issues in our focus groups. The choice to empower the advocates to drive and create the content is a novel and distinguishing feature of our model and was instrumental in facilitating trust and ensuring authenticity [36]. This approach led to content production that aligned with the personal brand of each advocate and facilitated connectedness to each community, which was identified in the focus groups as important. These findings that social media communications are favorably perceived when produced by reliable sources align with cultivation theory, as consistent social media messaging from trusted sources could cultivate a perception of credibility and reliability in health promotion.

### Comparison With Previous Work

There are multiple studies describing strategies for health promotion that engage community representatives in the dissemination of information to improve trust and facilitate the dissemination of social media messages [39], and that crossposting across multiple platforms is necessary to combat misinformation [40]. However, our study is the first to show how different content types behave across platforms, emphasizing the need to design public health content according to the platform and audience.

Previous studies have used impressions [41,42], reach [41], and engagements [41-45] or specific elements (eg, shares and comments) to assess the dissemination of social media messages. These metrics in raw form are a reflection of the number of followers and might not be an accurate representation of the reach of the messages, and there is limited evidence of how these metrics relate to subsequent behaviors; however, previous studies suggest that these are related. A study by Nadarzynski et al [38] showed that Facebook advertisements could lead to increased traffic to websites promoting chlamydia testing and related sexual health information, resulting in a 41% increase in test requests, showcasing how increases in engagement in social media can be associated with new behaviors.

Selecting the right metrics to evaluate outreach efforts must be individualized to the campaign’s goal. On the basis of our results, we suggest using a combination of reach and engagement metrics, maximizing the use of tools available in social media professional accounts, and evaluating these metrics regularly to facilitate tailoring to the audience [14,37]. Contrary to common recommendations by social media influencers and marketing experts, in our experience, more posts did not lead to more engagement and reach. This might reflect the relevance of the social media messages to the audiences.

In our experience, producing large amounts of content at a fast pace does not allow enough time to create intentional and relevant social media messages and might affect individuals’ recognition of the messages. According to Vakratsas and Ambler [46], a minimum of 3 exposures is often necessary for a social media message to have an impact, with diminishing returns after 10 to 20 exposures. The frequency of these social media messages and the complexity of the message also affect the effectiveness in promoting a new behavior or increasing interest in a product; according to a white paper by Facebook from 2016, one to two social media messages per week might be enough to generate recognition [47], which support the frequency selected for our messages.

### Limitations

Although our study explores many perspectives, 1 limitation was that our focus groups engaged a small number of individuals across the communities, which might affect the richness of our results and potential generalizability to other communities and geographic locations. Considering that our study was performed in Wisconsin, it is important to acknowledge that context factors affect the perceptions and potential interactions that individuals have with our social media posts. For example, in Wisconsin, vaccination against COVID-19 was free to all individuals, and there was no requirement for proof of residence or US citizenship to access these services. This resulted in less fear of deportation and lower mistrust in vaccination social media messages compared with what could be seen in other localities in the United States, where these services were only available to US residents.

Another limitation of our study is that our metrics do not account for reach and engagements after our posts were shared. In July 2021, Facebook analytics changed how it collected and presented metrics for pages; since then, only the metrics of the page are shown, not accounting for engagement on third-party pages, resulting in lower overall numbers. Similarly, our results do not reflect what happened outside the social media platforms, such as sharing information in group chats, WhatsApp, or SMS text messages, which are platforms frequently used by our communities [13]. To ensure uniformity of the metrics, all data for the study period were downloaded in March 2023 and used for all the analyses.

### Conclusions

This project created social media content that was medically accurate and culturally appropriate related to the COVID-19 pandemic and its physical and mental health consequences. One key aspect of our work is that we relied on building trustworthy relationships with partners and communities within and outside social media; this was achieved through the work of our advocates and team members who served as a connection with the communities. We identified that stories in videos, photos, or messages that contained only text created meaningful connections with individuals and provided trustworthy information with very encouraging results that we have been sharing with academicians and community organizations. However, our results showcase how analytics reports of the social media platforms (eg, share, likes, and views) remain inadequate metrics for measuring behavioral changes in the target audiences [48] and require the development of further population-health approaches to identify whether social media campaigns such as ours can translate on increased adoption and maintenance of health behavior and subsequently improved health outcomes. Future studies could explore whether social media engagements are associated with changes in beliefs and the acquisition of new health behaviors to promote health and well-being and how social media campaigns can build on community partnerships to address misinformation or disinformation at the population level in ways that are culturally appropriate and result in improved health outcomes.

## Appendix

Multimedia Appendix 1 Sample content created by the community advocates.

Multimedia Appendix 2 iCHECK-DH (Guidelines and Checklist for the Reporting on Digital Health Implementations) checklist.

Multimedia Appendix 3 Demographics of the followers of the community platforms.

## Abbreviations

ARPP: average reach per post
ERR: engagement rate per reached person
iCHECK-DH: Guidelines and Checklist for the Reporting on Digital Health Implementations
REDCap: Research Electronic Data Capture

## Data Availability

The datasets generated during and analyzed during this study are available from the corresponding author on reasonable request.
