# Supplementary material for: Implementation of a Social Media Strategy for Public Health Promotion in Black, American Indian or Alaska Native, and Hispanic or Latino Communities During the COVID-19 Pandemic: Cross-Sectional Study
Source: J Med Internet Res. 2024 Dec 10;26:e58581. doi: 10.2196/58581 (PMC11668992; doi:10.2196/58581)
Supplement: Multimedia Appendix 1 [file jmir_v26i1e58581_app1.pdf]

Multimedia Appendix 1:

A) Examples of Content/Posts for the Madison's Black Community

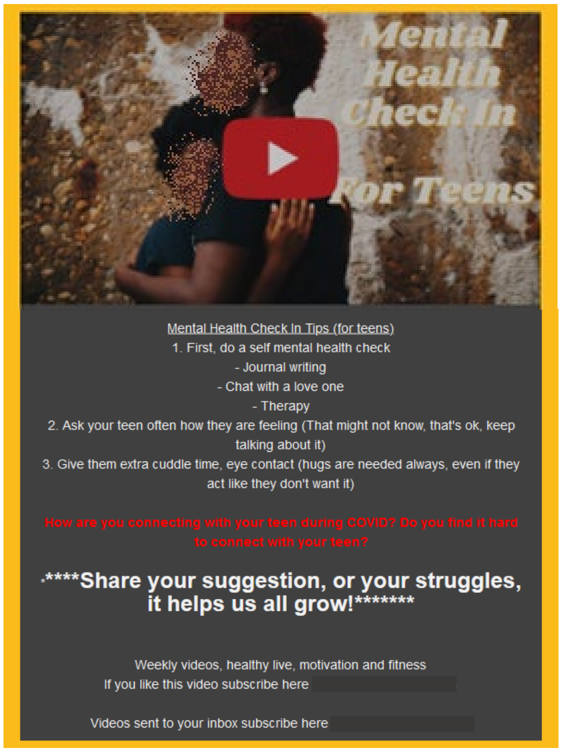

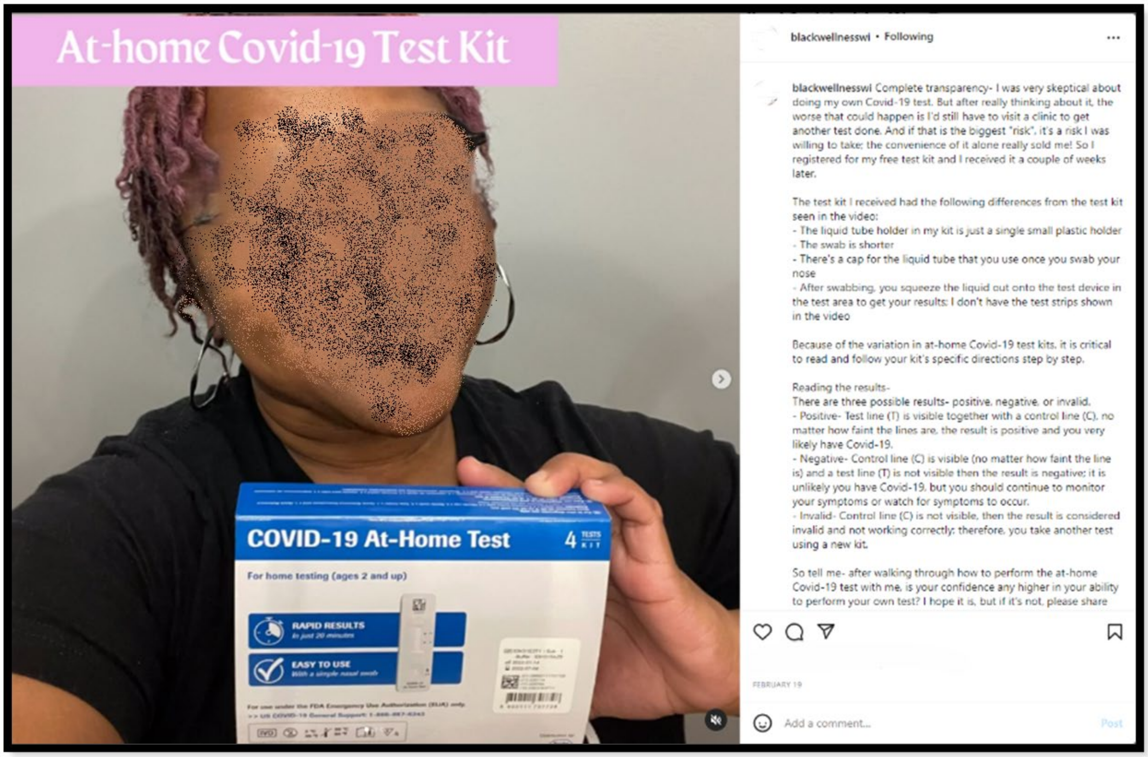

These posts were developed by staff at University of Wisconsin – Madison, for more information please contact corresponding author: Maria Mora Pinzon, MD at mcpinzon@medicine.wisc.edu.

B) Examples of Content/Posts for the Oneida Nation

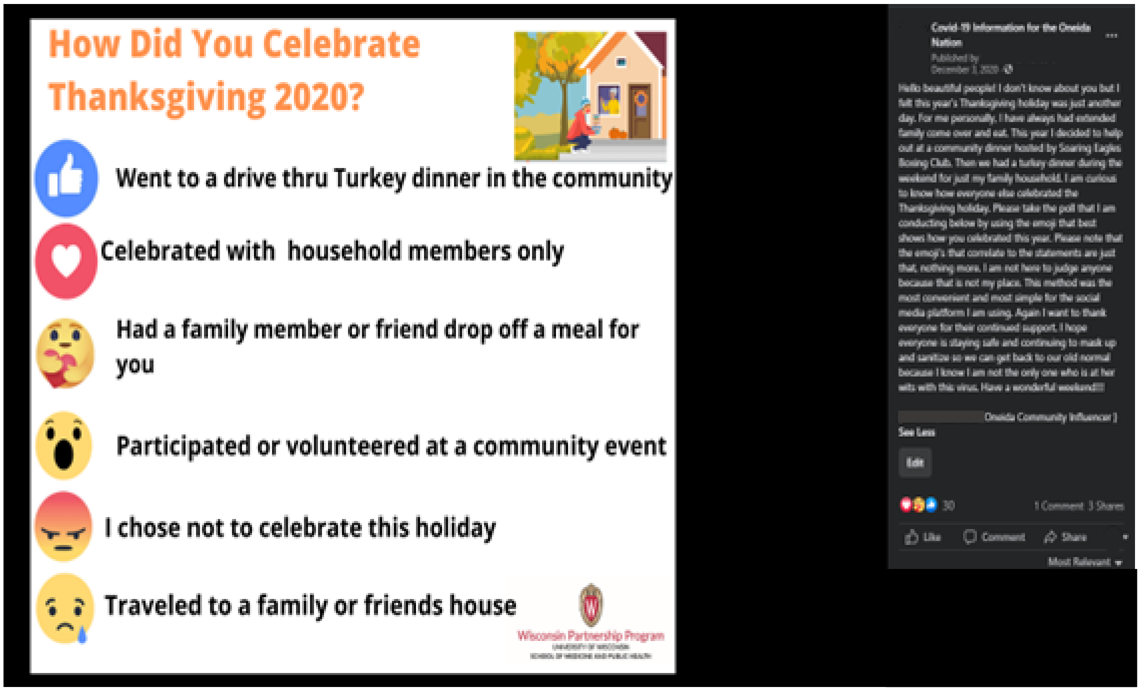

C) Examples of Content/Posts for the Madison's Latinx Community

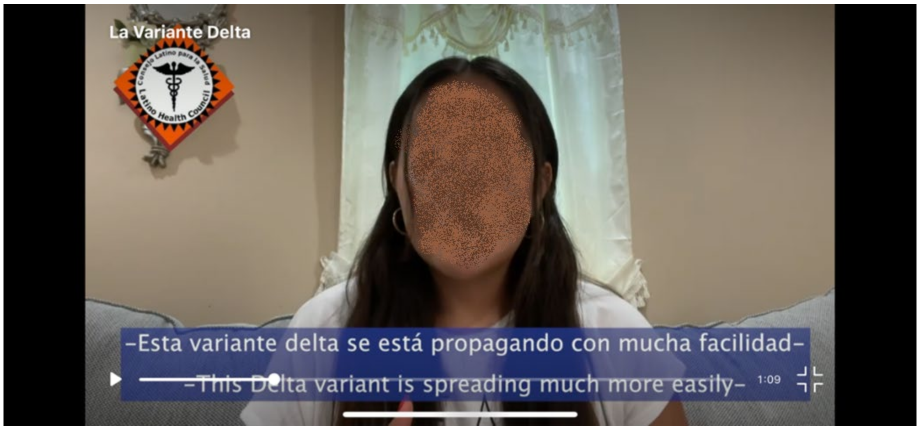

latinohealthcouncil La Variante Delta · La variante Delta es responsable por la mayoría de casos nueves en los Estados Unidos 🦠. Para protegerse a sí mismo y a su comunidad, usen su mascarilla, lávense las manos, mantengan su distancia, y hagan su cita para la vacuna 😷💉.

Para más información sobre la vacuna contra el Covid ——> https://publichealthmdc.com/espanol/coronavirus/vacuna-para-el-covid-19

#LatinoHealthCouncil #ConsejoLatino #Deltavariant #COVID19 #Coronavirus #Latinx #Wisconsin #Vaccine #Health #Vacuna #Explorepage

These posts were developed by staff at University of Wisconsin – Madison, for more information please contact corresponding author: Maria Mora Pinzon, MD at mcpinzon@medicine.wisc.edu.
