# Supplementary material for: Implementation of a Social Media Strategy for Public Health Promotion in Black, American Indian or Alaska Native, and Hispanic or Latino Communities During the COVID-19 Pandemic: Cross-Sectional Study
Source: J Med Internet Res. 2024 Dec 10;26:e58581. doi: 10.2196/58581 (PMC11668992; doi:10.2196/58581)
Supplement: Multimedia Appendix 3 [file jmir_v26i1e58581_app3.docx]

**Multimedia Appendix 3**

**A) Age & Gender Distribution of Followers of Black Community COVID-19 Pages**

**
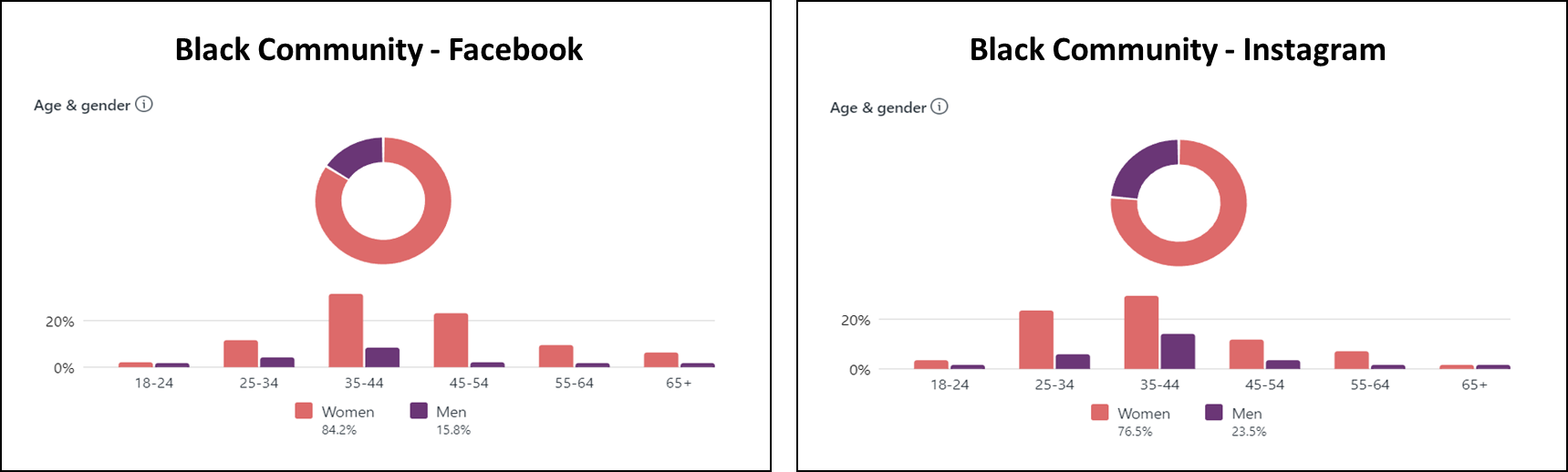
**

**B) Age & Gender Distribution of Followers of Oneida Nation COVID-19 Pages**

**
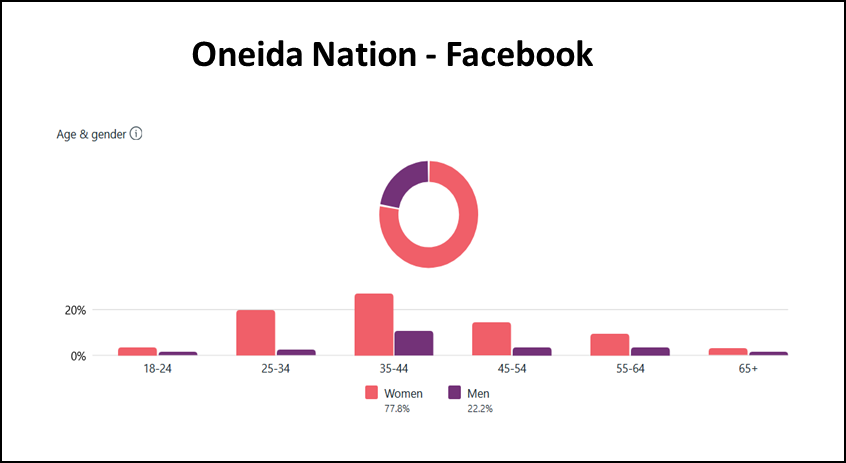
**

**C) Age & Gender Distribution of Followers of Latino Community COVID-19 Pages**

**
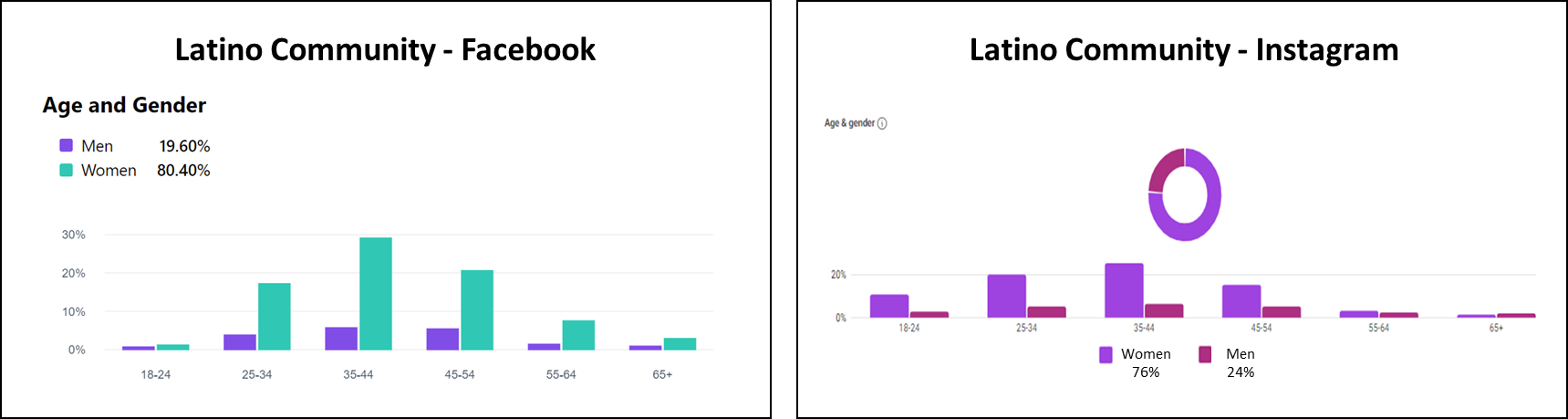
**
